# β-Glucans: Relationships between Modification, Conformation and Functional Activities

**DOI:** 10.3390/molecules22020257

**Published:** 2017-02-09

**Authors:** Qiang Wang, Xiaojing Sheng, Aimin Shi, Hui Hu, Ying Yang, Li Liu, Ling Fei, Hongzhi Liu

**Affiliations:** 1 Institute of Food Science and Technology, Chinese Academy of Agriculture Sciences, Beijing 100193, China; wangqiang06@caas.cn (Q.W.); s_ilence0101@126.com (X.S.); sam_0912@163.com (A.S.); littleh@vip.sina.com (H.H.); yingyang_umass@126.com (Y.Y.); liulicaas@126.com (L.L.); 2 Cornell University, Robert Frederick Smith School of Chemical and Biomolecular Engineering, Ithaca, NY 14850, USA; lingfei646@gmail.com

**Keywords:** β-glucans, modification, conformation transformation, functional activities

## Abstract

β-glucan is a type of polysaccharide which widely exists in bacteria, fungi, algae, and plants, and has been well known for its biological activities such as enhancing immunity, antitumor, antibacterial, antiviral, and wound healing activities. The conformation of β-glucan plays a crucial role on its biological activities. Therefore, β-glucans obtained from different sources, while sharing the same basic structures, often show different bioactivities. The basic structure and inter-molecular forces of polysaccharides can be changed by modification, which leads to the conformational transformation in solution that can directly affect bioactivity. In this review, we will first determine different ways to modify β-glucan molecules including physical methods, chemical methods, and biological methods, and then reveal the relationship of the flexible helix form of the molecule chain and the helix conformation to their bioactivities. Last, we summarize the scientific challenges to modifying β-glucan’s conformation and functional activity, and discuss its potential future development.

## 1. Introduction

**Figure 1:**
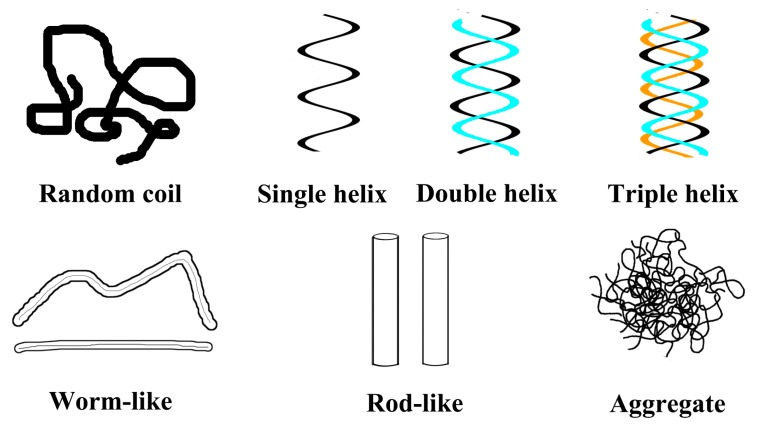
Scheme of possible polysaccharide chain conformations in aqueous solution.

**Figure 2:**
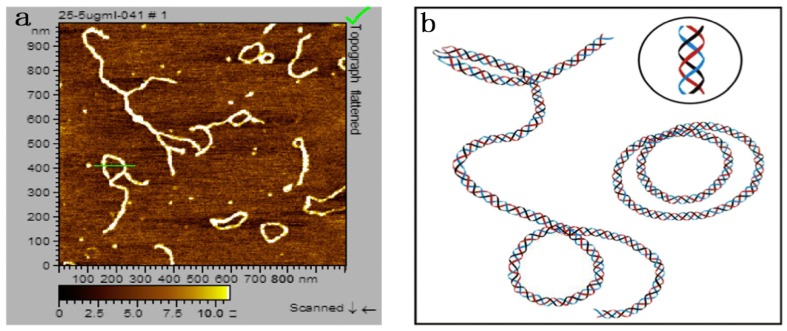
Atomic Force Microscope (AFM) image (**a**) and schematic representation of the lentinan solution at 25 °C (**b**). Adapted from [5], with permission from the American Chemical Society, Copyright 2008.

Polysaccharides, consisting of long chains of repeating sugar units, have been considered one of the most important classes of biopolymers, due to their biological activities such as enhancing immunity, antitumor, antibacterial, antiviral, and wound healing activities. The conformations of polysaccharides refer to the shape and size of polysaccharides in solution, including monosaccharide conformation, flexibility, spatial structure, and so on [1]. The conformations of polysaccharides in solution include random coil, single helix, double helix, triple helix, worm-like shape, rod-like shape, and aggregate (Figure 1). Because of their relatively small space steric hindrance and higher spin degree of freedom, linear α- or β-(1-3)-glucans show the random thread chain conformation. Moreover, β-(1-3)-glucans tend to form helix conformations because of the numerous hydrogen bonds on their side chains; for example, for lentinan composed of β-(1-6)-branched β-(1-3)-d-glucans, the Atomic Force Microscope (AFM) image (Figure 2) shows that lentinan exists as a helix in aqueous solution [2], curdlan is primarily composed of glucose units with linear -(1,3)-glycosidic linkages [3], and xyloglucan (XG) is a hemicellulose with a backbone formed by -(1,4)-d-glucan [4].

The structures, intra-molecular, and inter-molecular forces, such as hydrogen bonds in molecules or between molecules of polysaccharides, can remarkably affect their conformation. It is very possible to change the bioactivities of polysaccharides by modifying their structure [2,6,7]. Hence, a lot of physical, chemical, and biological methods have been studied to modify the conformations of polysaccharides for desired bioactivities. Among the polysaccharide family, β-glucan is widely found in bacteria, fungi, algae, and plants, and has been well known for its biological activities such as enhancing immunity, antitumor, antibacterial, antiviral, and wound healing activities. It was found that the basic structures of β-glucans from different sources are almost the same, but they often show different bioactivities. For example, curdlan and lentinan are both composed of β-(1-6)-glucan. However, lentinan has antitumor and antibacterial bioactivity, while curdlan does not. However, after sulphating modification, curdlan can inhibit the human immunodeficiency virus-1 (HIV-1) infection in H9 cells, because the conformation of sulfated curdlan is changed to triple helix [8]. The conformation is the key factor that decides the bioactivities of β-glucans. Therefore, in this review, we summarized the previous effort and progress made on the conformation transformations of β-glucans via different modification methods and shared our insights on future studies.

## 2. Effects of Physical Modification on the Conformation of β-Glucans

### 2.1. Ultrasonic Modification

Ultrasonic waves can cause degradation of the β-glucan molecular chains via breaking glucosidic bonds and initiating the oxidation-reduction reaction of free radicals. After ultrasonic modification, lower molecular weight fractions of β-glucans can be obtained [9], which not only show improved water solubility and the physiological function of β-glucans, but also exhibit conformation in solution.

For example, *Lentinus edodes* glucan with β-(1-6)-d branching in distilled water (0.2 wt %) was treated in a 33 kH ultrasonic device from 1 to 24 h, and the molecular weight of lentinan varied from 3.57 × 10^5^ Da to 28.3 × 10^5^ Da. The weight-averaged molecular weight (*Mw*) and radius of gyration (<*S*^2^>*z*^1/2^) of lentinan were determined by size-exclusion chromatography combined with multi-angle laser light scattering (SEC-LLS), while its intrinsic viscosity [η] was measured using viscometers. Meanwhile, these properties of lentinan in different mediums including 0.9% NaCl aqueous solution and dimethyl sulfoxide (DMSO) were compared. Analysis of *Mw*, [η], and <*S*^2^>*z*^1/2^ in terms of the known theory for worm-like chains [10] yielded 2240 ± 100 M/nm, and 100 ± 10 nm for molar mass per unit contour length (M_L_), and persistence length (*q*), respectively, corresponding with the theoretical data for triple-helical chains [2]. The results indicated that lentinan existed as a triple helix in 0.9% NaCl aqueous solution and as a single flexible chain in DMSO. Assays in vivo and in vitro against the growth of sarcoma 180 solid tumor as well as the colorimetric 3-(4,5-dimethylthiazol-2-yl)-2,5-diphenyltetra-zolium bromide (MTT) method for lentinan showed that the triple-helix sample exhibited a relatively high inhibition ratio. The triple-helix lentinan with *Mw* of 1.49 × 10^6^ exhibited the highest antitumor activity in vivo but the bioactivity of its single flexible chains almost disappeared. It was also found that the triple-helix conformation plays an important role in enhancing the antitumor effects of lentinan [11]. Usually, glucan with *Mw* less than 5 × 10^4^ Da cannot form a helix structure [2]. In this experiment, the *Mw* of the smallest fraction was 3.57 × 10^5^ Da. Therefore, the helix structure was mostly found in lentinan β-glucan, and the effect of ultrasonic treatment on the conformation of glucan was not obvious.

### 2.2. Irradiation Modification

β-glucan can also be modified through irradiation, including γ-ray, X-ray, and electron beam to cause physical and chemical changes in its structure [12]. Methacanon et al. [13] studied the effects of γ-ray modification on the conformation of β-glucan produced from *Ophiocordyceps* *dipterigena* BCC2073, and then investigated the relationship between the conformations of β-glucan and stimulated activities on interleukin-8 (IL-8). Results showed that the average molecular weight of irradiated samples significantly decreased as the irradiation dose increased. Specifically, the *Mw* of β-glucan decreased from 5.9 × 10^5^ Da to 5000 Da with the irradiation dose of γ-ray varying from 0 kGy to 100 kGy. Results suggested that β-glucans with a helix conformation (due to its low degree of branching) were less active in inducing the production of IL-8. For β-glucan with a molecular weight of 5000 Da, most of the chains seemed to be short and existed in the random coil form. It also exhibited the strongest ability to induce the production of IL-8. This result might be because smaller molecules had a higher chance of binding to receptors [13].

### 2.3. Microwave Modification

Microwave modification is also a very popular method for altering the conformation of β-glucan. The microwave energy may enhance the stretching of the molecular chain and finally affect the β-glucans’ physicochemical properties.

β-d-glucan (TM3a), extracted from the sclerotia of *Pleurotus tuber-regium*, was treated with microwaves to improve its solubility in water. Results showed that this method could lead to the complete dissolution of the β-d-glucan (TM3a) in 0.02% (*w*/*w*) NaN_3_ aqueous solution. The optimal microwave treatment conditions corresponded to 35 s at 765 W. Moreover, the solution properties of TM3a in water were studied systematically using size-exclusion chromatography combined with laser light scattering and viscometry at 25 °C (Table 1). The results indicated that TM3a existed in form of sphere-like conformation in 0.02% (*w*/*w*) aqueous NaN_3_ solution, and the spherical molecules of TM3a could be observed directly through transmission electron microscopy (TEM) [14].

### 2.4. Other Physical Modification Methods

**Figure 3:**
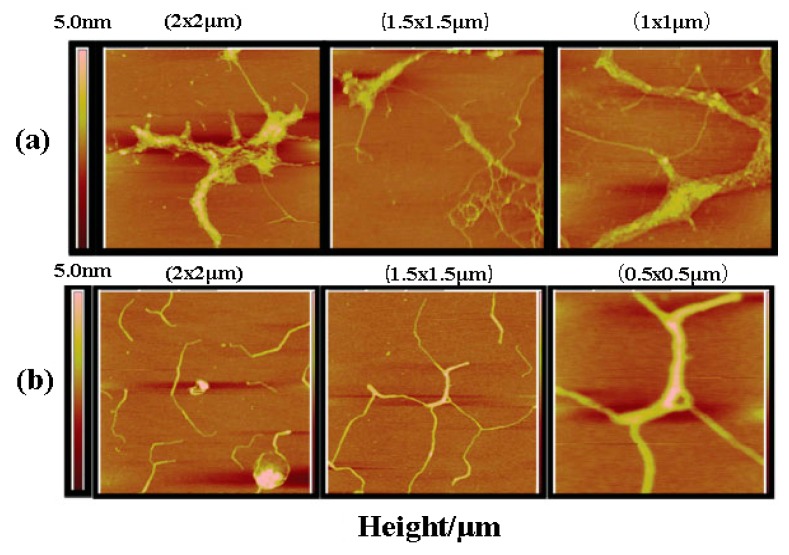
The AFM topology graph of *Pleurotus geesteranus* β-d-glucan at different magnifications at 4 °C (**a**) and 22 °C (**b**) (reproduced from [18] with permission from John Wiley and Sons, Copyright 2009).

Zhang et al. [18] investigated the effect of heating on the conformational change of *Pleurotus geesteranus* β-d-glucan. Heating the water solution of *Pleurotus geesteranus* β-d-glucan at the temperature range of 8–12.5 °C and 25–60 °C could result in the transition of their glucan conformations. The microstructures at 4 °C and 22 °C were imaged by AFM, and Figure 3 shows the large polymer bundles formed at 4 °C (Figure 3a) and the much smaller helical structures formed at 22 °C (Figure 3b). At 4°C, the aggregated conformers consist of bundles of polymers, when temperature goes to 22 °C, the side chain organization is weakened so that the polymer bundles dissociate into helical strands.

**Figure 4:**
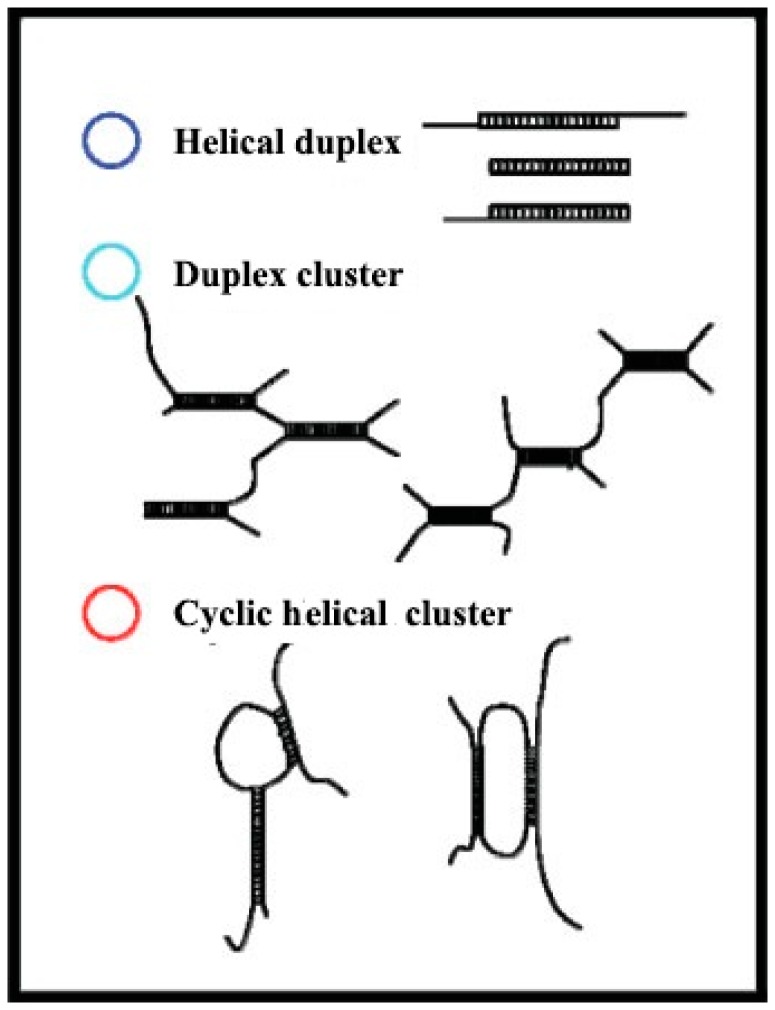
Schematic representation of the different types of helical structures of *Pleurotus geesteranus* β-d-glucan (reproduced from [18] with permission from John Wiley and Sons, Copyright 2009).

Figure 4 shows the schematic representation of how the helical strands are entangled to form different types of helical structures. The AFM topology images of *Pleurotus geesteranus* β-d-glucan at 4 °C and 22 °C reveal that the microstructure transition in the range from 8 °C to 12.5 °C is related to the disintegration of polymer bundles into small entwined helical strands.

High pressure microfluidization is a new physical method used in the modification of β-glucan. A solution is pumped and split into two microstreams, which then impact or collide against each other in a chamber, called the interaction chamber, where shear, turbulent, and cavitation forces are generated [19].

There are a few papers about the effect of high pressure microfluidization on the modification of β-glucan conformation at present, which tell us that high pressure microfluidization could decrease the *Mw* [20], change the microstructure [21], promote hydroscopicity, and enhance the solubility of polysaccharides [22,23,24]. Liu et al. [20] used ionic liquid as a solvent and treated with dynamic high pressure microfluidization to prepare water-soluble yeast β-glucan. Fourier Transform infrared spectroscopy (FTIR) and ^13^C-NMR spectra confirmed that water soluble β-glucan (WSG) is the polysaccharide with β-(1-3) glycosidic bonds. The chain conformation of water-soluble glucan in 0.1 M NaNO_3_ aqueous solutions at 25 °C were studied by laser light scattering and viscometry. The Mark-Houwink equation was established to be [*η*] =2.09 × 10^−2^ *Mw*^0.63^ (cm^3^·g^−1^). On the basis of conformational parameters calculated from the wormlike cylinder model, the water-soluble glucan existed as a semi-stiff chain in aqueous solution [20].

## 3. Effects of Chemical Modification on the Conformation of β-Glucan

### 3.1. Carboxymethylated Modification

The preparation of carboxymethyl β-glucan usually uses chloroacetic acid or sodium chloroacetate as a substrate. β-Glucan reacts with the substrate in alkaline 2-acetone solution. Zhang et al. [25] extracted six different types of mushroom (1-3)-β-glucans from the clerotia of *Pleurotus tuber-regium* using the hot alkali method. The extracts have molecular weights ranging from 1 × 10^4^ Da to 42 × 10^4^ Da. They were then carboxymethylated to generate the water-soluble derivatives with molecular weights varying from 2.08 × 10^4^ Da to 53.2 × 10^4^ Da. In general, the carboxymethylated β-glucan has higher water solubility, and higher in vivo and in vitro anti-tumoral activity than the original β-glucan. From an ELISA assay of cytokines, carboxymethylated β-glucan could induce the generation of tumor necrosis factor α in mouse plasma stimulated by lipopolysaccharide. It is possible that the expanded flexible chains of these novel carboxymethylated β-glucans are responsible for their in vivo and in vitro antitumor activities.

A water-insoluble glucan (*Poria cocos* sclerotium β-glucan, PCSG) isolated from the fresh sclerotium of *Poria cocos* was carboxymethylated to obtain its water-soluble derivative coded as C-PCSG (Carboxymethylated *Poria cocos* sclerotium β-glucan). Wang et al. [26] studied the conformation of C-PCSG solutions. The conformation equations and parameters are shown in Table 1. The results indicate that C-PCSG exists as a relatively extended flexible chain in the 0.2 M NaCl aqueous solution, while PCSG exists as a random coil in DMSO. Therefore, The C-PCSG sample possesses more stiff chains compared with its original glucan (PCSG). Thus, the introduction of the carboxymethyl groups on β-glucan could improve the water solubility significantly and enhance the stiffness of the chains.

Wang et al. [27] studied the effects of carboxymethylation on the water solubility of *Sacchammyces cerevisiae* β-glucan. Their average *Mw* is around 10^5^ Da and their conformation in solution is mostly in the form of a helix. Moreover, carboxymethyl glucan of *S. cerevisiae* did not show cytotoxic or genotoxic effects, or any effect on the cell viability [28]. A linear water-insoluble glucan was extracted successfully from the fruit body of *Ganoderma lucidum*. Five water-soluble derivatives were prepared from the water-insoluble glucan by sulfation, carboxymethylation, hydroxyethylation, hydroxypropylation, and methylation, respectively. There was no obvious aggregation for the derivatives in either Me_2_SO or 0.9% aqueous NaCl solution. GL-IV-I exists as a compact coil chain in Me_2_SO at 25 °C, whereas its derivatives exist as relatively expanded flexible chains in 0.9% aqueous NaCl solution at 25 °C, owing to the steric hindrance of the substituted groups [29].

### 3.2. Sulfated Modification

Sulfated modification was another method to improve the water solubility of β-glucan through the introduction of hydrophilic groups (sulfate radical). Sulfated β-glucans have many advantages such as good water solubility, high yield, and some important biological activities including anticoagulant, antithrombotic, and anti-cancer, and their inflammatory and immune responses [30,31]. Williams et al. [32] used DMSO, urea, and concentrated sulfuric acid to produce sulfate derivatives of insoluble β-(1-3)-d-glucan, which was isolated from *S. cerevisiae.* Two polymer peaks were resolved by aqueous high-performance size exclusion chromatography (HPSEC) with on-line multi-angle laser light scattering (MALLS) photometry and differential viscometry. This study found that sulfated β-glucan had many advantages such as macrophages activation, stimulated bone marrow, antitumor therapeutic activity, and immunoprophylaxis which will modify the course of experimental infectious diseases.

Zhang et al. [33] investigated sulfated modification of a water-insoluble β-d-glucan from the sclerotia of *Pleurotus tuber-regium* and studied the conformation changes of the modified β-d-glucan. Through sulfated modification, the *Mw* could vary from 5.76 × 10^4^–77.4 × 10^4^ to 6.0 × 10^4^–64.8 × 10^4^ as the degree of substitution (DS) ranged from 1.14 to 1.74. The *Mw* and the intrinsic viscosity were also measured by size-exclusion chromatography combined with laser light scattering (SEC-LLS) and viscometry, respectively, in phosphate buffer solution (PBS) at 37 °C. The conformation equations and parameters of the sulfated modification of β-d-glucan are shown in Table 1. Sulfonation of the TM8 fractions, from the sclerotia of *P. tuber-regium*, can help improve the water solubility and chain stiffness of the β-d-glucan. Based on the analysis of the parameters, sulfonated TM8 has more expanding flexible chains than that of the original TM8 in DMSO. The higher stiffness of its chain may result from the polyelectrolytic effect caused by the sulfate groups substituted on the backbone. Liu et al. [10] modified the β-d-glucan extracted from *S. cerevisiae* by the sulfated method and studied the water solubility and the conformations of the sulfate derivatives. The conformation equations and molecular parameters of sulfated β-d-glucan were obtained by LLS and viscometry (Table 1). The results indicated that the sulfated β-d-glucan was relatively extending the flexible chains in the aqueous solution.

### 3.3. Phosphorylated Modification

The level of polysaccharides’ bioactivities is closely related to their chemical composition, molecular weight, chain conformation, and water-solubility [34]. Williams et al. [35] introduced a phosphorylated modification method for the solubilization of a micro-particulate β-d-glucan from *S. cerevisiae*. Molecular-weight averages, polydispersity, and intrinsic viscosity were determined, and two polymer peaks were resolved. Peak 1 (*Mw* = 3.57 × 10^6^) represents about 2% of the total polymers, while peak 2 (*Mw* = 1.10 × 10^5^) comprises about 98% of the polymers. In solution, the glucan-phosphate compound self-associated in the form of a triple helix and expressed excellent solubility and bioactivity.

Müller et al. [36] extracted β-glucan from *S. cerevisiae* using various protic acids including hydrochloric acid, acetic acid, formic acid, and phosphoric acid. Then these micro-particulates were employed as the starting materials for the production of (1-3)-β-d-glucan phosphate. The results indicated that the treatments using different acids had remarkable influences on the average *Mw*, molecular size, polydispersity, and intrinsic viscosity of β-d-glucan. (1-3)-β-d-glucan isolated from *Poria cocos* was phosphorylated to obtain a series of phosphorylated derivatives [15,16]. Chen et al. [15] and Huang et al. [16] investigated the conformation and the anti-tumor activity of the phosphorylated derivatives. The conformation equations and molecular parameters of the phosphorylated derivatives are shown in Table 1. In 0.15 M NaCl aqueous solution, these phosphorylated derivatives exist in the form of relatively extended flexible chains. Compared with the original (or untreated) β-d-glucan, the phosphorylated derivatives show increased water-solubility and chain stiffness, which could be explained by the introduction of phosphate groups on the main chains. Moreover, all the phosphorylated derivatives exhibit significantly stronger anti-tumor activities than that of the unphosphorylated derivatives. This result also suggested that the solubility and expanded chain conformation would possibly improve the anti-tumor activity.

A water-insoluble (1-3)-β-d-glucan from *Poria cocos* mycelia was fractionated, followed by phosphorylation with H_3_PO_4_ in LiCl/Me_2_SO containing urea to synthesize water-soluble phosphorylated derivatives. Their structures and chain conformations were investigated by FTIR, NMR, SEC-LLS and viscometry. The Mark-Houwink equation for the phosphorylated derivative in 0.15 M aqueous NaCl at 30 °C was established to be [η] = 2.87 × 10^−3^ *M_W_*^0.86^ ^± 0.02^. On the basis of the conformation parameters, the phosphorylated derivative of (1-3)-β-d-glucan existed as a semi-stiff chain in 0.15 M NaCl aqueous solution. The phosphorylated derivatives exhibited significant in vivo and in vitro anti-tumor activities against sarcoma 180 tumor cells compared with the unphosphorylated derivatives, suggesting that the effect of solubility and expanded chains on the improvement of the anti-tumor activities was not negligible. In addition, the relatively moderate molecular weight (5′ × 10^4^ to 50′ × 10^4^) was favorable for the enhancement of the anti-tumor activities for polysaccharide [16].

## 4. Effects of Biological Modification on the Conformation of β-Glucan

β-Glucanase is commonly used to decrease the *Mw* and increase the water solubility and bioactivity of β-glucan. β-glucan extracted from oat was partially degraded by β-glucanase for different periods of time [17]. The average molecular weight and intrinsic viscosity were measured. The conformation equations and molecular parameters are shown in Table 1. The results indicated that enzyme treatment could make the conformation of oat β-glucan vary from a semi-flexible chain to an extended random coil.

Duan et al. [37] hydrolyzed yeast β-(1-3)-glucan isolated from *Trichoderma* strain LE02 using β-1,3-glucanase. They found that fractions in the hydrolysates with *Mw* larger than 3 × 10^4^ accounted for 66.2% of the total sample and that the water solubility was also improved. Moreover, anti-tumor and antioxidant activities of the enzymatic hydrolysis samples had significantly increased. There were few studies on the conformation of yeast β-glucan through enzymatic hydrolysis, and more research on this is desired [1].

## 5. Effects of Conformation Transformation on the Functional Activity of β-Glucan

### 5.1. Effects of Backbone Flexibility on the Function Activity

The backbone flexibility of polysaccharides is determined by hydrogen bonding and electrostatic repulsive forces. After modification, such as sulphating, the backbone flexibility can be enhanced and can lead to improved functional activity [38]. Mueller et al. [39] characterized the structure of the polysaccharides’ receptor on the surface of organic precursors, and found that the chain with higher stiffness could bind with the receptor on the cell surface easily and showed higher bioactivity. Chen et al. [15] studied the relationship of pachymaran conformation to its antitumor activity. They found that after phosphate esterification, β-(1-3)-d-glucan in *Poria coco*s showed flexible chains in 0.15 M NaCl solution and could resist the tumor sarcoma 180 effectively.

**Figure 5:**
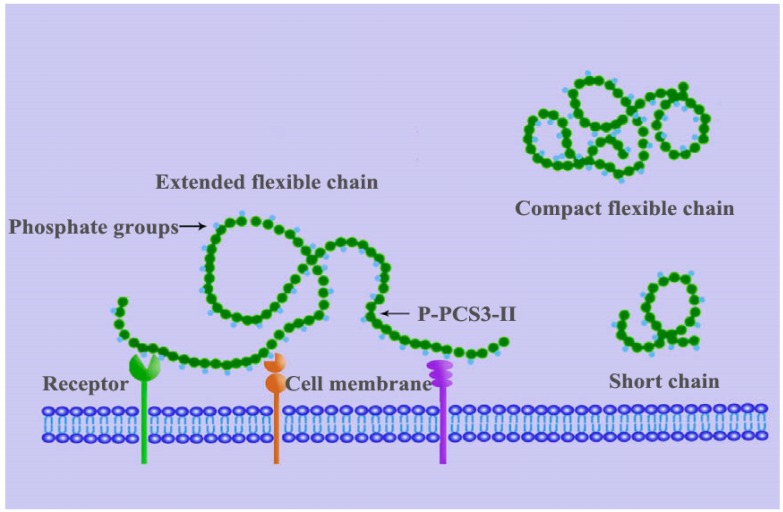
Schematic depiction of the interaction between the phosphorylated pachymaran P-PCS3-II chain and receptors on the immune cell (reprinted from *Carbohydr. Polym.*, *78*, Chen, X.; Xu, X.; Zhang, L.; Zeng, F., Chain conformation and anti-tumor activities of phosphorylated (1→3)-β-d-glucan from *Poria cocos*, pp. 581–587, Copyright (2009), with permission from Elsevier [15]).

It was concluded that the flexible chain can enhance the antitumor activity of polysaccharides. As shown in Figure 5, Chen et al. [15] also proposed a model to describe the interaction between the polysaccharide and receptors on the immune cell membrane. There were several kinds of polysaccharide receptors on the immune cells, and phosphate group in P-PCS3-II (the phosphorylated precipitate obtained from *Poria cocos*) could bind the receptors with high affinity [40]. β-(1-3)-d-glucan in *Poria coco*s P-PCS3-II with relatively expanded chains and relatively high molecular weight has more chance to collide and bind more receptors. This leads to the enhancement of the proliferation, differentiation of immune cells, and the production of cytokines such as interferons, interleukins, TNF-α, and other tumor cells. Such enhancement can intensify the immune reaction which inhibits the proliferation of the tumor [41,42].

A water-soluble hyper branched (1-4)-d-glucan with a degree of branching (DB) value of 35% was successfully used to synthesize the carboxymethyl polysaccharides, the sulfated polysaccharides, and the phosphated polysaccharides were dissolved in 2-propanol or DMSO solutions [31]. A SEC-MALLS-Vis-RI combination instrument was used to characterize their molar weights and chain conformations successfully. The results illustrated that (1-4)-d-glucan adopted a condensed coil conformation, and the derivatives adopted more extended flexible coil conformations, indicating that the chain extensibility depends on the type of substituent groups and the degree of substitution. Anti-tumor activity assays indicated that the sulfated polysaccharides and phosphated polysaccharides exhibited prominent inhibition against H-22 tumor cells both in vitro and in vivo; the sulfated polysaccharides showed higher activity in vitro while phosphated polysaccharides showed higher activity in vivo. Therefore, the polysaccharides with negative charges and proper molecular weights possessed higher anti-tumor activity [31].

### 5.2. Effects of Helix Conformation on the Function Activity

It is known that the polysaccharides with a β-helix structure usually have stronger bioactivity [43]. For example, lentinan has significant antitumor activity. X-ray analysis showed that it was composed of β-(1-3)-d-glucan with β-helix structures [11]. On the other hand, the polysaccharides with random structures have lower bioactivities. If the lentinan was dissolved in DMSO, the bioactivity would be dismissed because the conformation is changed. The antitumor activity (sarcoma 180) of lentinan with a different conformation showed that lentinan composed of a helix chain had a higher antitumor activity in comparison with those that have a single strand random thread chain. Once the helix chain was destroyed, the antitumor activity decreased significantly [11].

Pachymaran, whose structure is similar to lentinan except for having the side chain of β-(1-6)-glucan, does not show any antitumor activity. After periodate oxidation and Smith degradation, the β-(1-6) side chain of pachymaran was removed, and it gained antitumor activity. X-ray analysis of pachymaran without the β-(1-6) side chain showed that it had the helix structure. In addition, both schzophyllan and small nuclear bacteria dextran extracted from mushroom with helix structures had the antitumor activity [44]. On the contrary, Saitô et al. [45] purified the β-(1-3)-d-glucan with a single strand chain, and the product showed higher bioactivity than that of the polysaccharides with helix structures. Therefore, further study is necessary to determine the relationship between the chain structures (random chain or the helix chain) and the bioactivities of polysaccharides.

## 6. Conclusions

Recently, the conformation of β-glucan has been a hot topic of research, but only a few references about the conformation transformation of β-glucan can be found in the literature. Therefore, researchers should further study the conformation transformation of β-glucan and modified β-glucan to find the relationship between the conformations and bioactivities of β-glucan, in order to design functionalized structures of β-glucan with higher bioactivities in the future [46].

Modification can significantly affect the primary structure and spatial conformation of β-glucan, and different modification methods show dissimilar effects on the glucan structures. For instance, physical modification affects the conformation of the modified polysaccharide through changing its spatial structure instead of the primary structure; chemical modification can introduce groups on the main chain or side chain, which leads to the changes of both the primary structure and spatial structure. Therefore, it is very important to study the effects of the modification methods on the glucan structure, and to determine the optimal modification methods for achieving β-glucans with higher bioactivities. For example, Li et al. [12] found that polydeoxyadenylic acid (poly(dA)) could react with single chains of lentinan (s-LNT) and form a novel composite (5.3 × 10^5^ Da) with a stiff conformation through hydrogen bonding. However, the triple helical lentinan (t-LNT) could not produce such a composite. β-Glucan can be modified by physical, chemical, and biological methods, but some methods cannot be adopted in industry because of the treatment capacity, equipment, reagent safety, and so on. Meanwhile, there are still only a few studies on the conformation changes of β-glucan before and after physical modification. Hence, the relationship between the conformation and bioactivity of β-glucan applied in industrial fields still needs to be investigated systematically.

In addition, the conformation transformation of modified β-glucan and the effect of specific technological or processing parameters on these transformations should be further studied. Li et al. [12] studied the conformation transition of lentinan β-glucan in water and DMSO. Results indicated that the glucan in 0.9% NaCl aqueous solution existed as triple-helical chains with high rigidity while in DMSO it existed as single-flexible chains, and the conformation transition from single-flexible chains to helix chains occurred in a narrow range from 80 wt % to 85 wt % of DMSO aqueous solution. During the modifications, some new functional groups would be linked to the branches of β-glucan, which would lead to the changes of the bioactivities. However, the reasons and mechanisms of this variation are still unclear as both the primary structure and spatial conformation may affect the bioactivities of β-glucan, and it is very difficult to differentiate their contributions. It is generally recognized that polysaccharides with helix structures have higher bioactivities [47], but Saitô et al. [45] verified the β-(1-3)-d-glucan with a single strand chain showed higher bioactivity than the polysaccharides with a helix structure. Hence, the relationship of polysaccharide conformation and bioactivity still needs further study.

## Figures and Tables

**Table 1:** Conformations and correlation parameters of modified β-glucan.

Glucan	Modification Methods	Solution	*Mw* Range	Conformations and Correlation Parameters	Ref.
*Pleurotus tuber-regium* β-glucan	Microwave	0.02% NaN_3_	8.20 × 10^5^~4.88 × 10^6^	Sphere-like chain. [η] = 3.02 × 10^−2^ *Mw*^0.44 ± 0.1^ (cm^3^·g^−1^). <*S*^2^>*z*^1/2^ = 8.32 × 10^−2^ *Mw*^0.38 ± 0.2^(nm)	[14]
Yeast β-glucan	Sulfate	0.2 M·NaCl	2.22~8.78 × 10^4^/5.06~8.78 × 10^4^	Semi-stiff chain. [η] = (5.03 ± 0.31) × 10^−3^ *Mw*^0.84 ± 0.03^ (cm^3^·g^−1^), Yamakawa-Fujii-Yoshizaki (YFY) worm-like cylinder model, M_L_ = 646 nm^−1^, *q* = 5.1 nm, *C_∞_* = 16.33. <*S*^2^>*z*^1/2^ = (2.33 ± 0.40) × 10^−3^ *Mw*^0.62 ± 0.02^ (nm), Kratky-Porod (KP) worm-like chain model, M_L_ = 596 nm^−1^, *q* = 8.7 nm	[10]
*Poria cocos* β-glucan	Phosphory-lated	0.15 M·NaCl	2.6 × 10^4^~26.9 × 10^4^	Relatively extended flexible. [η] = 6.61 × 10^−3^ *Mw*^0.73^ (cm^3^·g^−1^), worm-like cylinder model, M_L_ = 818 nm^−1^, *q* = 3.0 nm, *C_∞_* = 5.2	[15]
*Poria cocos* mycelia α-glucan	Phosphory-lated	0.15 M·NaCl	1.96 × 10^4^~15.6 × 10^4^	Semi-stiff chain. [η] = 2.87 × 10^−3^ *Mw*^0.86 ± 0.02^ (mL·g^−1^), M_L_ = 780 ± 40 nm^−1^, *q* = 6.0 ± 1 nm, *C_∞_* = 15.1 ± 1	[16]
Oat β-glucan	Enzyment		2.2 × 10^3^~2.1 × 10^5^	Random coil. [η] = 1.06 × 10^−2^ *Mw*^0.86^	[17]

[*η*]: Intrinsic viscosity; *Mw*: Molecular weight; *<S^2^>_z_^1/2^*: Mean-square radius of gyration; M_L_: molar mass per unit contour length; *q*: Persistence length of molecular chains; *C_∞_*: Characteristic ratio of polymer molecules.

## References

[1] Wang Q., Liu H.Z., Zhong K. (2011). Review on the relationship of polysaccharide molecular chain conformation changes and bioactivities. Curr. Biotechnol..

[2] Li S., Xu S., Zhang L.N. (2010). Advances in conformations and characterizations of fungi polysaccharides. Acta Polym. Sin..

[3] McIntosh M., Stone B.A., Stanisich V.A. (2005). Curdlan and other bacterial (1→3)-d-glucanss. Appl. Microbiol. Biotechnol..

[4] Todaro S., Sabatinoa M.A., Mangione M.R., Picone P., Giacinto M.L.D., Bulone D., Dispenza C. (2016). Temporal control of xyloglucan self-assembly into layered structures by radiation-induced degradation. Carbohydr. Polym..

[5] Wang X., Xu X., Zhang L. (2008). Thermally Induced Conformation Transition of Triple-Helical Lentinan in NaCl Aqueous Solution. J. Phys. Chem. B.

[6] Zhang Y., Li S., Wang X., Zhang L., Cheung P.C.K. (2011). Advances in Lentinan: Isolation, structure, chain conformation and bioactivities. Food Hydrocoll..

[7] Zhang M., Kim J.A. (2012). Effect of molecular size and modification pattern on the internalization of water soluble β-(1→3)-(1→4)-glucan by primary murine macrophages. Int. J. Biochem. Cell Biol..

[8] Jagodzinski P.P., Wiaderkiewicz R., Kurzawski G., Kloczewiak M., Nakashima H., Hyjek E., Yamamoto N., Uryu T., Kaneko Y., Posner M.R. (1994). Mechanism of the Inhibitory Effect of Curdlan Sulfate on HIV-1 infection in vitro. Virology.

[9] Li J.B., Li L., Li B., Chen L., Huang G.X. (2006). Advances in ultrasonic degradation of polysaccharide. Sci. Technol. Food Ind..

[10] Liu X.Y. (2007). Studies on the Preparation, Conformation and Immunobiological Activity of β-d-Glucan in *Saccharomyces cerevisiae*. Ph.D Thesis.

[11] Zhang L., Li X., Xu X., Zeng F. (2005). Correlation between antitumor activity, molecular weight, and conformation of Lentinan. Carbohydr. Res..

[12] Li Y.J., Ha Y.M., Fan P., Wang F., Li A. (2009). Application of radiation technology in molecular modification of polysaccharides: A review. Food Sci..

[13] Methacanon P., Weerawatsophon U., Tanjak P., Rachtawee P., Prathumpai W. (2011). Interleukin-8 stimulating activity of low molecular weight β-glucan depolymerized by γ-irradiation. Carbohydr. Polym..

[14] Tao Y., Xu W. (2008). Microwave-assisted solubilization and solution properties of hyperbranched polysaccharide. Carbohydr. Res..

[15] Chen X., Xu X., Zhang L., Zeng F. (2009). Chain conformation and anti-tumor activities of phosphorylated (1→3)-β-d-glucan from *Poria cocos*. Carbohydr. Polym..

[16] Huang Q., Zhang L. (2011). Preparation, chain conformation and anti-tumor activities of water-soluble phosphated (1→3)-α-d-glucan from *Poria cocos* mycelia. Carbohydr. Polym..

[17] Roubroeks J.P., Andersson R., Mastromauro D.I., Christensen B.E., Åman P. (2001). Molecular weight, structure and shape of oat (1→3),(1→4)-β-d-glucan fractions obtained by enzymatic degradation with (1→4)-β-d-glucan 4-glucanohydrolase from *Trichoderma reesei*. Carbohydr. Polym..

[18] Zhang M. (2010). Heating-induced conformational change of a novel β-(1→3)-d-glucan from *Pleurotus geestanus*. Biopolymers.

[19] Kasaai M.R., Charlet G., Paquin P., Arul J. (2003). Fragmentation of chitosan by microfluidization process. Innov. Food Sci. Emerg. Technol..

[20] Liu H.Z., Li Y.N., Gao J., Shi A.M., Liu L., Hu H., Putri N., Yu H.W., Fan W., Wang Q. (2006). Effects of microfluidization with ionic liquids on the solubilization and structure of β-d-glucan. Int. J. Biol. Macromol. Carbohydr. Polym..

[21] Kan M., Zhang Y., Liu C.M., Liu W., Wang J. (2010). Effect of Dynamic High Pressure Microfluidization on Molecular Weight Distribution and Functional Groups of Hemicellulose B Fractions Purified from Soybean Dregs Dietary Fibers. Food Sci..

[22] Lagoueyte N., Paquin P. (1998). Effects of microfluidization on the functional properties of xanthan gum. Food Hydrocoll..

[23] Wang Y., Li D., Wang L.J., Xue J. (2011). Effects of high pressure homogenization on rheological properties of flaxseed gum. Carbohydr. Polym..

[24] Bonilla J., Atarés L., Vargas M., Chiralt A. (2012). Effect of essential oils and homogenization conditions on properties of chitosan-based films. Food Hydrocoll..

[25] Zhang M., Cheung P.C.K., Zhang L., Chiu C.M., Ooi V.E.C. (2004). Carboxymethylated β-glucan from mushroom sclerotium of *Pleurotus tuber-regium* as novel water-soluble anti-tumor agent. Carbohydr. Polym..

[26] Wang Y., Zhang L. (2006). Chain conformation of carboxymethylated derivatives of (1→3)-β-d-glucan from *Poria cocos* sclerotium. Carbohydr. Polym..

[27] Wang M., Ding X. (1998). The physiochemical properties and the conformation in the solution of modified yeast glucan—CMG. Chin. J. Biochem. Mol. Biol..

[28] Magnani M., Calliari C.M., de Macedo F.C., Mori M.P., de Syllos Cólus I.M., Castro-Gomez R.J.H. (2009). Optimized methodology for extraction of (1→3)(1→6)-β-d-glucan from *Saccharomyces cerevisiae* and in vitro evaluation of the cytotoxicity and genotoxicity of the corresponding carboxymethyl derivative. Carbohydr. Polym..

[29] Wang J.G., Zhang L. (2009). Structure and chain conformation of five water-soluble derivatives of a β-d-glucan isolated from *Ganoderma lucidum*. Carbohydr. Res..

[30] Wijesinghe W.A.J.P., Jeon Y.J. (2012). Biological activities and potential industrial applications of fucose rich sulfated polysaccharides and fucoidans isolated from brown seaweeds: A review. Carbohydr. Polym..

[31] Cong C., Wu W., Xu X. (2014). Chain conformation and anti-tumor activity of derivatives of polysaccharide from *Rhizoma Panacis Japonici*. Carbohydr. Polym..

[32] Williams D.L., Pretus H.A., McNamee R.B., Jones E.L., Ensley H.E., William Browder I., Di Luzio N.R. (1991). Development, physicochemical characterization and preclinical efficacy evaluation of a water soluble glucan sulfate derived from *Saccharomyces cerevisiae*. Immunopharmacology.

[33] Zhang M., Zhang L., Wang Y., Cheung P.C.K. (2003). Chain conformation of sulfated derivatives of β-glucan from sclerotia of *Pleurotus tuber-regium*. Carbohydr. Res..

[34] Zjawiony J.K. (2004). Biologically active compounds from *Aphyllophorales* (Polypore) fungi. J. Nat. Prod..

[35] Williams D.L., McNamee R.B., Jones E.L., Pretus H.A., Ensley H.E., Browder I.W., Di Luzio N.R. (1991). A method for the solubilization of a (1→3)-β-d-glucan isolated from *Saccharomyces cerevisiae*. Carbohydr. Res..

[36] Müller A., Ensley H., Pretus H., McNamee R., Jones E., McLaughlin E., Chandley W., Browder W., Lowman D., Williams D. (1997). The application of various protic acids in the extraction of (1→3)-β-d-glucan from *Saccharomyces cerevisiae*. Carbohydr. Res..

[37] Duan H.K. (2007). Anti-Tumor Activity of Enzymatic Hydrolysates of β-1,3-Glucan from *Saccharomyces cerevisiae*. Master’s Thesis.

[38] Carlucci M.J., Ciancia M., Matulewicz M.C., Cerezo A.S., Damonte E.B. (1999). Antiherpetic activity and mode of action of natural carrageenans of diverse structural types. Antivir. Res..

[39] Mueller A., Raptis J., Rice P.J., Kalbfleisch J.H., Stout R.D., Ensley H.E., Browder W., Williams D.L. (2000). The influence of glucan polymer structure and solution conformation on binding to (1→3)-d-glucan receptors in a human monocyte-like cell line. Glycobiology.

[40] Rice P.J., Kelley J.L., Kogan G., Ensley H.E., Kalbfleisch J.H., Browder I.W., Williams D.L. (2002). Human monocyte scavenger receptors are pattern recognition receptors for (1→3)-β-d-glucan. J. Leukoc. Biol..

[41] Thornton B.P., Vĕtvicka V., Pitman M., Goldman R.C., Ross G.D. (1996). Analysis of the sugar specificity and molecular location of the beta-glucan-binding lectin site of complement receptor type 3 (CD11b/CD18). J. Immunol..

[42] Vetvicka V., Thornton B.P., Ross G.D. (1996). Soluble beta-glucan polysaccharide binding to the lectin site of neutrophil or natural killer cell complement receptor type 3 (CD11b/CD18) generates a primed state of the receptor capable of mediating cytotoxicity of iC3b-opsonized target cells. J. Clin. Investig..

[43] Misaki A., Kakuta M., Sasaki T., Tanaka M., Miyaji H. (1981). Studies on interrelation of structure and antitumor effects of polysaccharides: Antitumor action of periodate-modified, branched (1→3)-β-d-glucan of *auricularia*
*auricula-judae*, and other polysaccharides containing (1→3)-glycosidic linkages. Carbohydr. Res..

[44] Yu Z., Ming G., Kaiping W., Zhixiang C., Liquan D., Jingyu L., Fang Z. (2010). Structure, chain conformation and antitumor activity of a novel polysaccharide from *Lentinus edodes*. Fitoterapia.

[45] Saitô H., Yoshioka Y., Uehara N., Aketagawa J., Tanaka S., Shibata Y. (1991). Relationship between conformation and biological response for (1→3)-β-d-glucan in the activation of coagulation factor G from limulus amebocyte lysate and host-mediated antitumor activity. Demonstration of single-helix conformation as a stimulant. Carbohydr. Res..

[46] Kagimura F.Y., da Cunha M.A., Barbosa A.M., Dekker R.F., Malfatti C.R. (2015). Biological activities of derivatized d-glucans: A review. Int. J. Biol. Macromol..

[47] Li X.L. (2004). Correlation of Conformation Transition to Bioactivities of Lentinan from Lentinusedoes. Master’s Thesis.

